# KIAA0247 suppresses the proliferation, angiogenesis and promote apoptosis of human glioma through inactivation of the AKT and Stat3 signaling pathway

**DOI:** 10.18632/oncotarget.13527

**Published:** 2016-11-23

**Authors:** Ying Tan, Ning Huang, Xiang Zhang, Jiangang Hu, Si Cheng, Li Pi, Yuan Cheng

**Affiliations:** ^1^ Department of Neurosurgery, The Second Affiliated Hospital of Chongqing Medical University, Chongqing, China; ^2^ Department of Orthopaedics, The Second Affiliated Hospital of Chongqing Medical University, Chongqing, China

**Keywords:** KIAA0247, glioma, cell proliferation, cell apoptosis, angiogenesis

## Abstract

Gliomas are the most common and aggressive type of primary adult brain tumors. Although KIAA0247 previously is a speculated target of the tumor suppressor gene, little is known about the association between KIAA0247 and glioma. In this study, we clearly demonstrate that KIAA0247 expression is decreased in glioma and was negatively correlated with the histologic grade. Overexpression of KIAA0247 in glioma cells inhibits proliferation, angiogenesis and promoted apoptosis of human glioma cells *in vitro*. In contrast, knockdown of KIAA0247 increases the proliferation, angiogenesis and decreases apoptosis of these cells. In a tumor xenograft model, overexpression of KIAA0247 suppresses tumor growth of glioma cells *in vivo*, while KIAA0247 knockdown promotes the tumor growth. Mechanistically, overexpression of KIAA0247 is able to inhibit phosphorylation of AKT and Stat3 in glioma cells, resulting in inactivation of the AKT and Stat3 signaling pathways, this ultimately decreases the expression of PCNA, CyclinD1, Bcl2 and VEGF. Collectively, these data indicate that KIAA0247 may work as a tumor suppressor gene in glioma and a promising therapeutic target for gliomas.

## INTRODUCTION

Glioma is the most common malignant tumor in human central nervous system, which incidence is about 7.2 per 100,000 population annually [1]. Their invasive growth makes complete tumor resection very difficult leading to high lethality [2]. Maximal tumor resection followed by radiotherapy and chemotherapy is the current standard of care for glioma patients [3]. Although adjuvant TMZ chemotherapy improves the clinical survival rate of glioblastoma, the prognosis is poor, with the median survival rate less than 15 months [4, 5]. Due to the failure of conventional strategies, understanding of the molecular mechanisms of glioblastoma will be help for treating glioblastoma and the delivery of tumoricidal agents. KIAA0247, a novel gene, contains six exons and encodes a 303–amino acid protein [6]. It previously was discovered as a speculated target of the tumor suppressor, p53, because of the p53 response element located in the promoter region [7, 8]. Some researchers found the expression of KIAA0247 in ovarian tumors was inverse association with ovarian tumor stage [6]. Earlier study showed low KIAA0247 RNA level in feces was strongly correlated with colorectal tumor size and a poor survival time [9]. However, the function of KIAA0247 in glioma is still largely unknown.

Signal transducer and activator of transcription 3 (Stat3) is known to be activated by growth factors, many cytokine and some oncogenes [10, 11] and upregulation of phosphorylated Stat3 was reported to be involved in activating multiple oncogenes which promotes cell cycle progression, anti-apoptosis, angiogenesis, migration and invasion [12–15]. AKT is also activated by diverse stimuli, such as growth factors, extracellular matrix components and hormones [16]. This activation finally leads to the apoptosis inhibition [17], cell cycle progression [18], tumor growth [19], impairment of G1 and G2 cycle arrest [20], and angiogenesis [21]. Many previous evidences have reported that phosphorylated AKT and phosphorylated Stat3 are significantly involved in glioma pathogenesis [22–24] and decreasing of phosphorylated AKT and phosphorylated Stat3 plays an important role in suppressing tumors [25–28].

In the present study, we detected the expression of KIAA0247 in glioma specimens and glioma cell lines and validated the relationships between KIAA0247 expression and the clinicopathologic characteristics of glioma patients. Furthermore, the effects of KIAA0247 on proliferation, angiogenesis and apoptosis were examined after overexpressing or downexpressing this gene in glioma cells. Based on our findings, we demonstrated that KIAA0247 suppressed glioma cell proliferation and angiogenesis and promoted apoptosis via inactivation of AKT and Stat3 signaling and their downstream targets. These evidences clarify the anti-tumor function of KIAA0247 in glioma, and are help for exploring the underlying mechanisms of KIAA0247 in regulating development of glioma.

## RESULTS

### Downexpression of KIAA0247 in human glioma tissues

**Figure 1:**
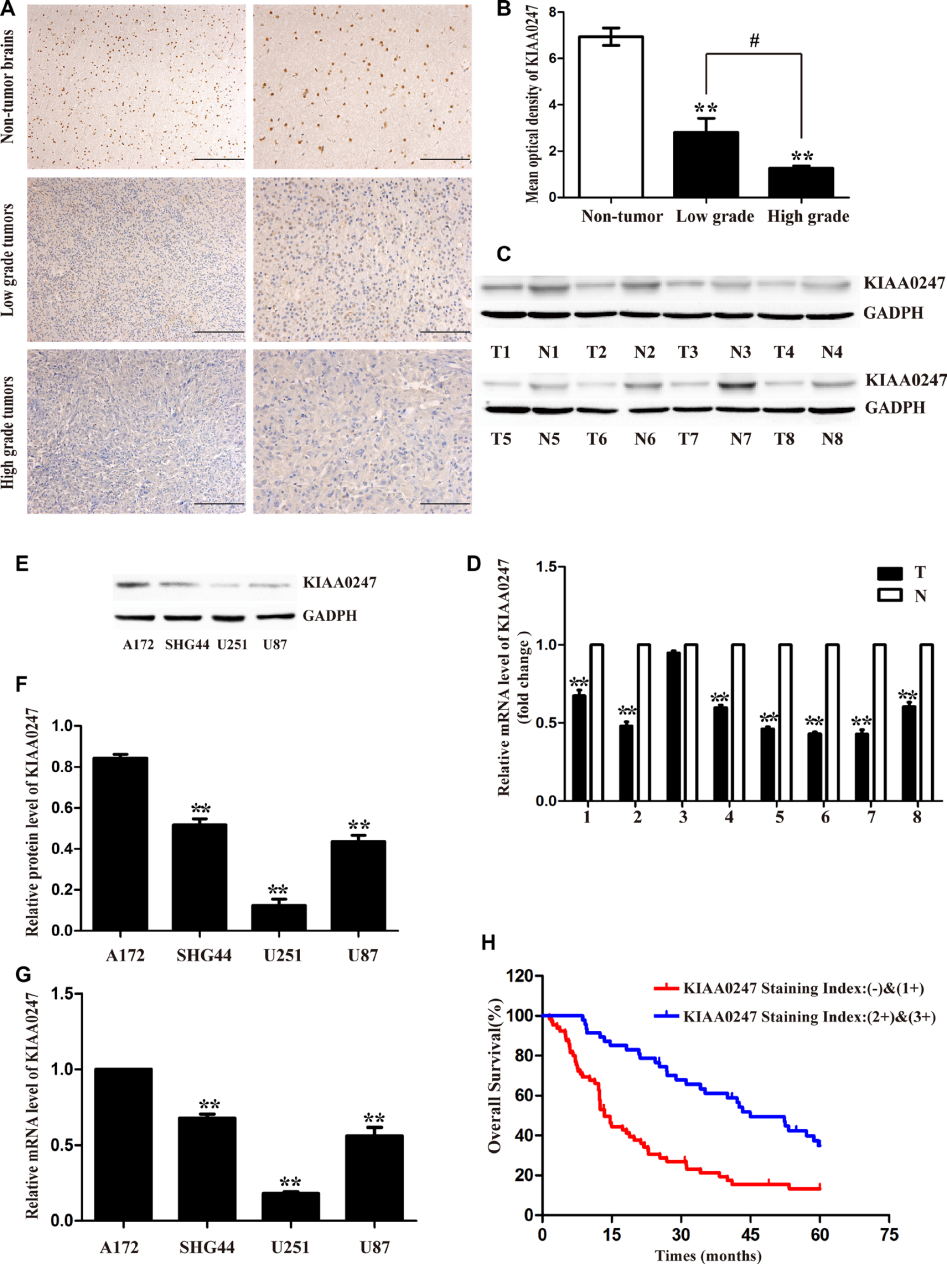
Low KIAA0247 expression predicted a worse prognosis for patients with glioma (**A**). Representative images of KIAA0247 immunostaining in non-tumor brain tissues, low grade-glioma tissues (WHO I-II) and high-grade glioma tissues (WHO III-IV). Low power (20×) scale bars: 100 μm, high power (40×) scale bars: 50 μm. (**B**) Statistical quantification of the mean optical density in non-tumor brain tissues, low-grade glioma tissues, and high-grade glioma tissues (**P < 0.01 versus non-tumor brain tissues; #*P* < 0.05 between the high-grade group and low-grade group). (**C**) Western blot shows KIAA0247 expression was lower in 8 pairs of predominately glioma tissues (T) compared to paired adjacent non-tumor tissues (N). (**D**) qRT-PCR analysis of KIAA0247 mRNA in 8 glioma tissues and paired adjacent non-tumor tissues (**P < 0.01 versus paired adjacent non-tumor tissues). (**E**, **F**) Western blot analysis of endogenous expression of KIAA0247 in four glioma cell lines (**P < 0.01 versus A172). (**G**) qRT-PCR analysis of endogenous KIAA0247 expression in four glioma cell lines (**P < 0.01 versus A172). (**H**) Kaplan-Meier analysis of overall survival based on KIAA0247 expression in 112 patients with glioma (*P* < 0.01).

To assess the protein expression levels of KIAA0247 in glioma tissues, immunohistochemistry (IHC) was used to analysis 123 glioma specimens (Supplementary Table S1). The data confirmed KIAA0247 was low-expression in glioma tissues compared with that in non-tumor brains, and the high-grade glioma presented a lower expression level of KIAA0247 than low-grade glioma (Figure 1A and 1B). There were no statistical significances in gender, age according to the staining results (Supplementary Table S2). We further analyzed the expression levels of KIAA0247 in eight glioma tissues (T) and their adjacent paired normol tissues (N) by qRT-PCR (Figure 1D) and Western blot analysis (Figure 1C and Supplementary Table S3). KIAA0247 mRNA level in glioma samples was significantly lower than that in the adjacent normal tissue. Similarly, the protein expression level of KIAA0247 in glioma tissues was a marked decrease when compared with the paired tissues. Furthermore, A Kaplan-Meier survival curve and log-rank tests revealed that patients with low KIAA0247 expression had a close relationship with poor overall survival (Figure 1H).

### KIAA0247 suppresses cell proliferation of glioma cells

**Figure 2:**
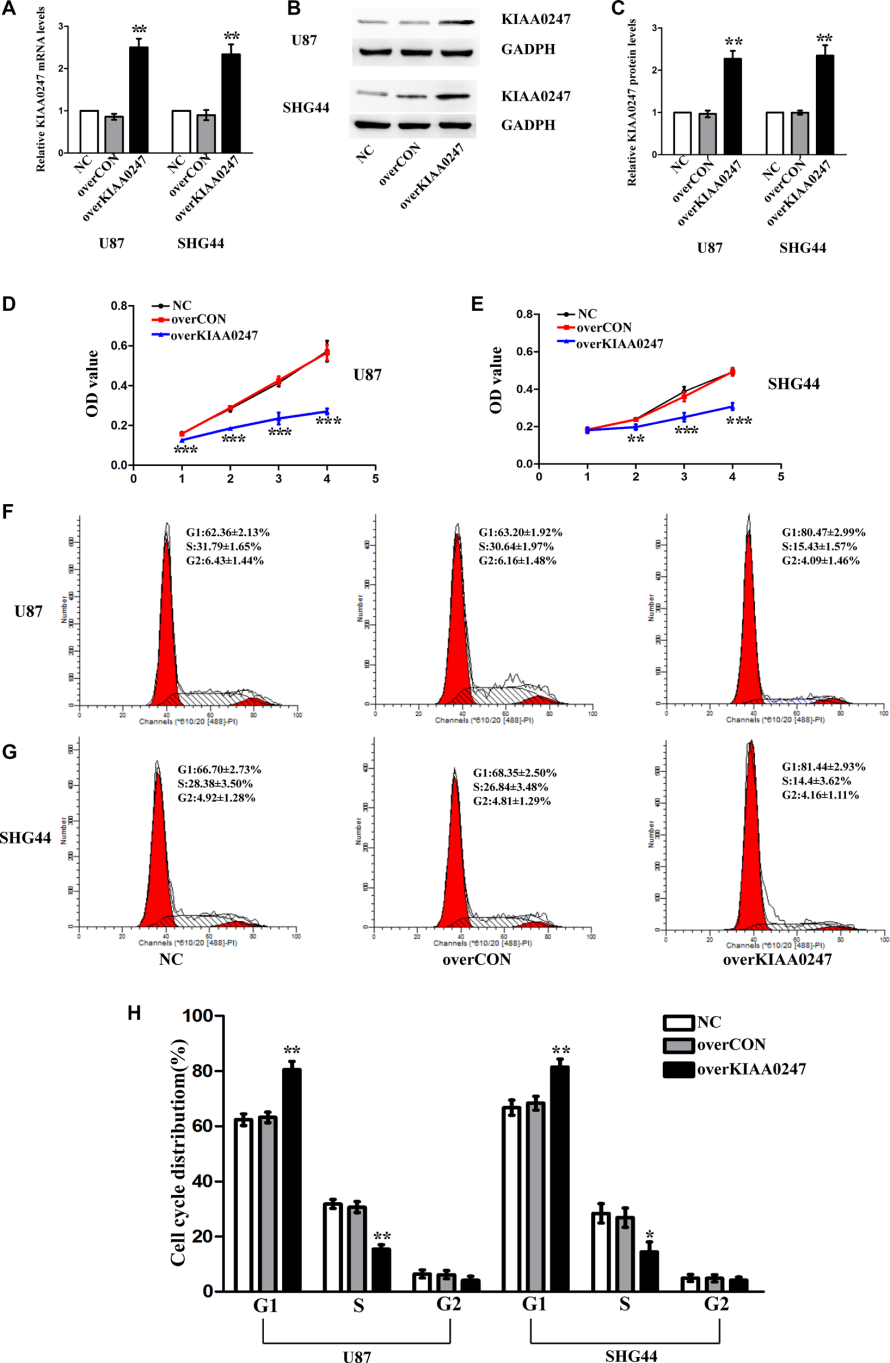
Upregulation of KIAA0247 inhibited proliferation of glioma cells (**A**) qRT- PCR was conducted to determine the mRNA levels of KIAA0247 in tin U87 and SHG44 cells. (**B**, **C**) Expression of KIAA0247 in U87 and SHG44 cells was analyzed by western blot. (**D**, **E**) Cell proliferation was detected in U87 and SHG44 cells by CCK-8 assay. (**F**–**H**) Cell cycle distributions were tested in in U87 and SHG44 cells by flow cytometry. NC: blank control cells; overCON: transfected with empty vectors; overKIAA0247: transfected with KIAA0247 vectors. Data were based on at least 3 independent experiments, and shown as the mean ± SD. (*P < 0.05, **P < 0.01, ***P < 0.001, compared with NC).

**Figure 3:**
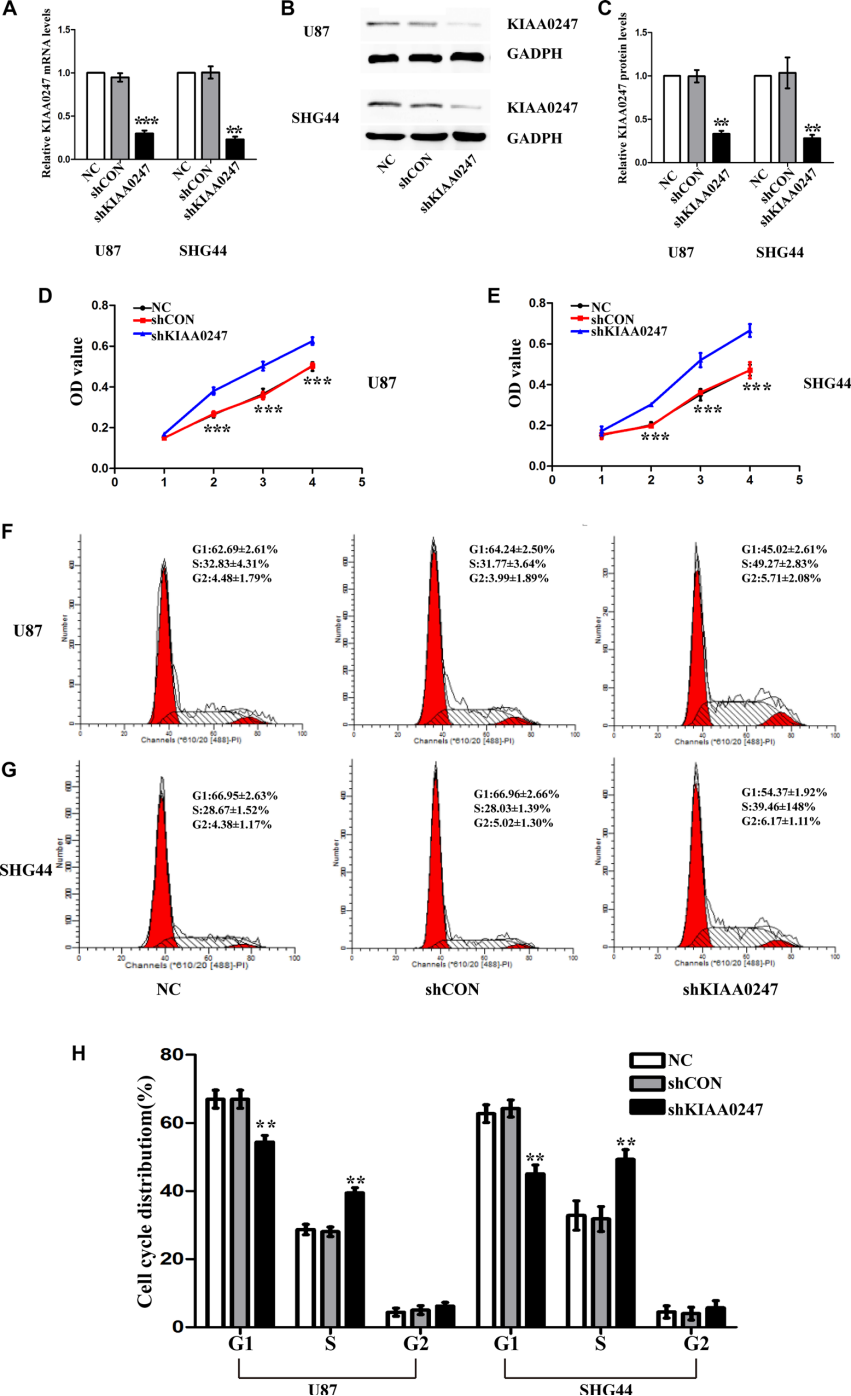
Knockdown KIAA0247 promoted proliferation of glioma cells (**A**) qRT- PCR was conducted to determine the mRNA levels of KIAA0247 in tin U87 and SHG44 cells. (**B**, **C**) Expression of KIAA0247 in U87 and SHG44 cells was analyzed by western blot. (**D**, **E**) Cell proliferation was detected in U87 and SHG44 cells by CCK-8 assay. (**F**–**H**) Cell cycle distributions were tested in in U87 and SHG44 cells by flow cytometry. NC: blank control cells; shCON: transfected with control shRNA; shKIAA0247 transfected with shKIAA0247 vectors. Data were based on at least 3 independent experiments, and shown as the mean ± SD. (**P < 0.01, ***P < 0.001 compared with NC).

U87, SHG44 glioma cell lines were selected for further investigations used in subsequent experiments after mRNA (Figure 1G) and protein detection (Figure 1E and 1F). We established KIAA0247-overexpressing U87 cell and SHG44 cell (U87 overKIAA cell and SHG44 overKIAA cell) and control cells (U87 overCON cell and SHG44 overCON cell) as well as KIAA-knockdown cells (U87 shKIAA cell and SHG44 shKIAA cell) and the control cells (U87 shCON cell and SHG44 shCON cell), as described in Materials and Methods. Both the levels of KIAA0247 mRNA and protein in overKIAA0247 (Figure 2A–2C) and ShKIAA0247 cells (Figure 3A–3C) were confirmed by qRT-PCR and western blot.

The effect of KIAA0247 on U87 and SHG44 cells growth was investigated by CCK8 assays. OverKIAA0247 cells grew significantly more slowly than the controls (Figure 2D and 2E). On the other hand, KIAA0247 knockdown markedly increased the U87 and SHG44 cells growth (Figure 3D and 3E). These data suggested that KIAA0247 has a negative effect on the growth of human glioma cells.

### KIAA0247 induces apoptosis and G1/S-phase arrest of glioma cells

**Figure 4:**
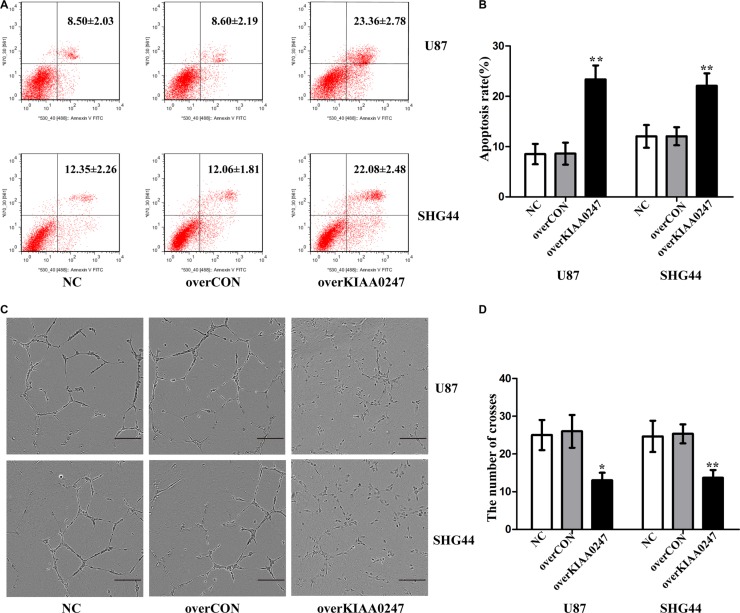
Upregulation of KIAA0247 induced apoptosis of glioma cells and suppressed tubule formation of HUVEC (**A**, **B**) Cell apoptosis was analyzed by Annexin V/PI staining. (**C**) Tube formation by HUVECs cultured in supernatants from U87 and SHG44 cells (10×), scale bars: 200 μm. (**D**) Image J software was applied to evaluate tubule crosses in (C) Data were based on at least 3 independent experiments, and shown as the mean ± SD. (*P < 0.05, **P < 0.01, compared with NC).

**Figure 5:**
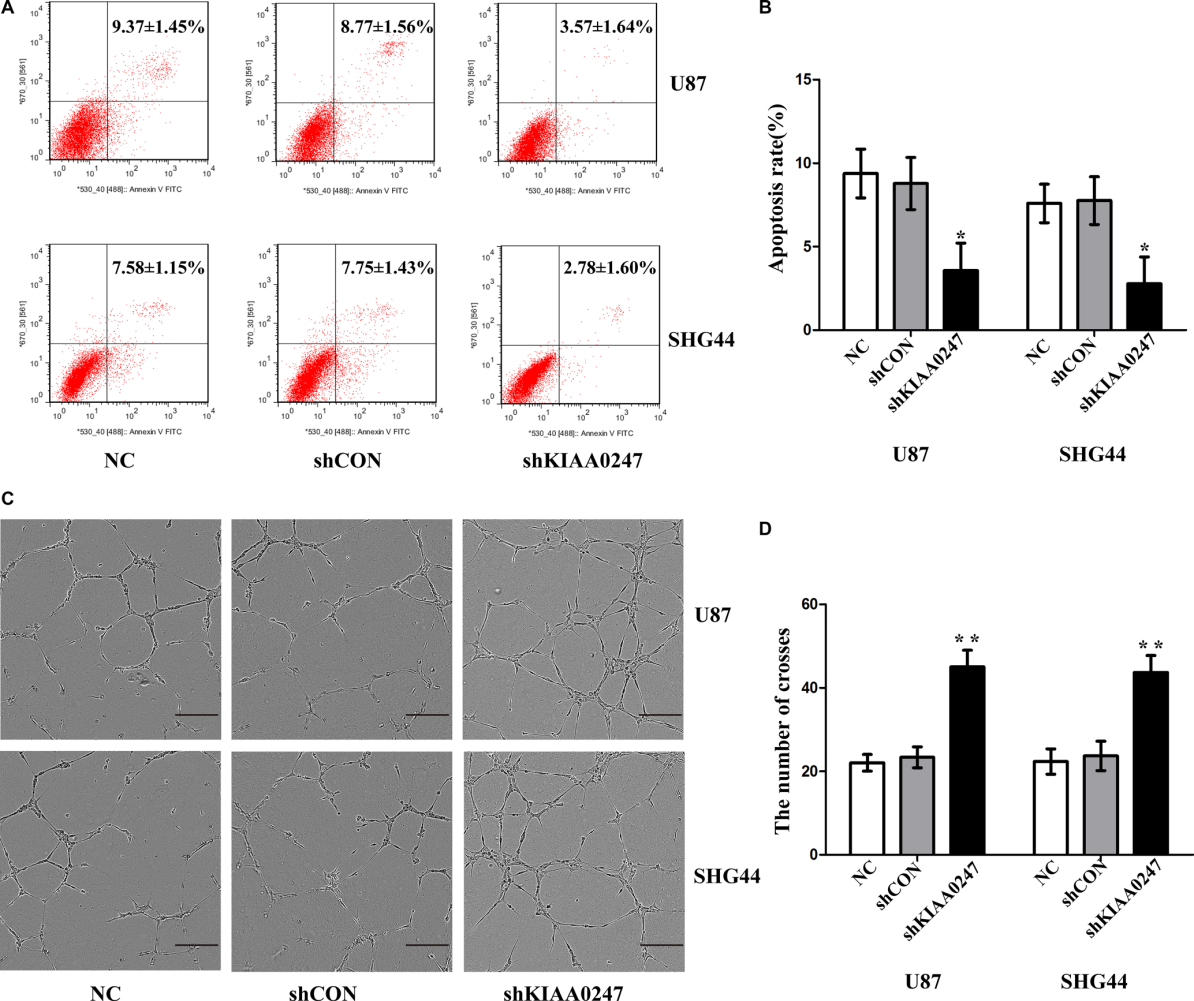
Knockdown KIAA0247 suppressed apoptosis of glioma cells and promoted tubule formation of HUVEC (**A**, **B**) Cell apoptosis was analyzed by Annexin V/PI staining. (**C**) Tube formation by HUVECs cultured in supernatants from U87 and SHG44 cells (10×), scale bars: 200 μm. (**D**) Image J software was applied to evaluate tubule crosses in (C) Data were based on at least 3 independent experiments, and shown as the mean ± SD. (*P < 0.05, **P < 0.01, compared with NC).

To examine the role of KIAA0247 in the cell cycle of glioma cells, cell cycle distribution was assessed in U87 and SHG44 cells by flow cytometry. The results indicated that the percentages of G1 phase in overKIAA cells were higher than that in the controls, whereas the percentages of S phase in overKIAA cells were lower than that of the controls (Figure 2F–2H). However, we found the percentages of G1 phase in shKIAA0247 cells were decreased and the percentages of S phase in cells were increased (Figure 3F–3H). In addition, we investigated the effects of KIAA0247 on apoptosis in glioma cells. Apoptotic cells were significantly elevated in overKIAA cells compared with the control cells (Figure 4A and 4B). In contrast, KIAA0247 knockdown reduced the rate of early and late apoptosis in glioma cells (Figure 5A and 5B). Thus, these results indicated that KIAA0247 suppresses cell cycle progression and promotes apoptosis of glioma cells.

### KIAA0247 inhibits tumor angiogenesis *in vitro*

Tube-formation assay was performed to determine the effect of KIAA0247 on angiogenesis of glioma cells. As a result, the number of crosses were markedly decreased in overKIAA0247 cells compared with the controls (Figure 4C and 4D), whereas knockdown of KIAA0247 increased number of crosses (Figure 5C and 5D). Collectively, these results implied that KIAA0247 attenuated the glioma cell-induced angiogenesis of endothelial cells *in vitro*.

### KIAA0247 inhibits AKT and STAT3 signaling pathway in glioma cells

**Figure 6:**
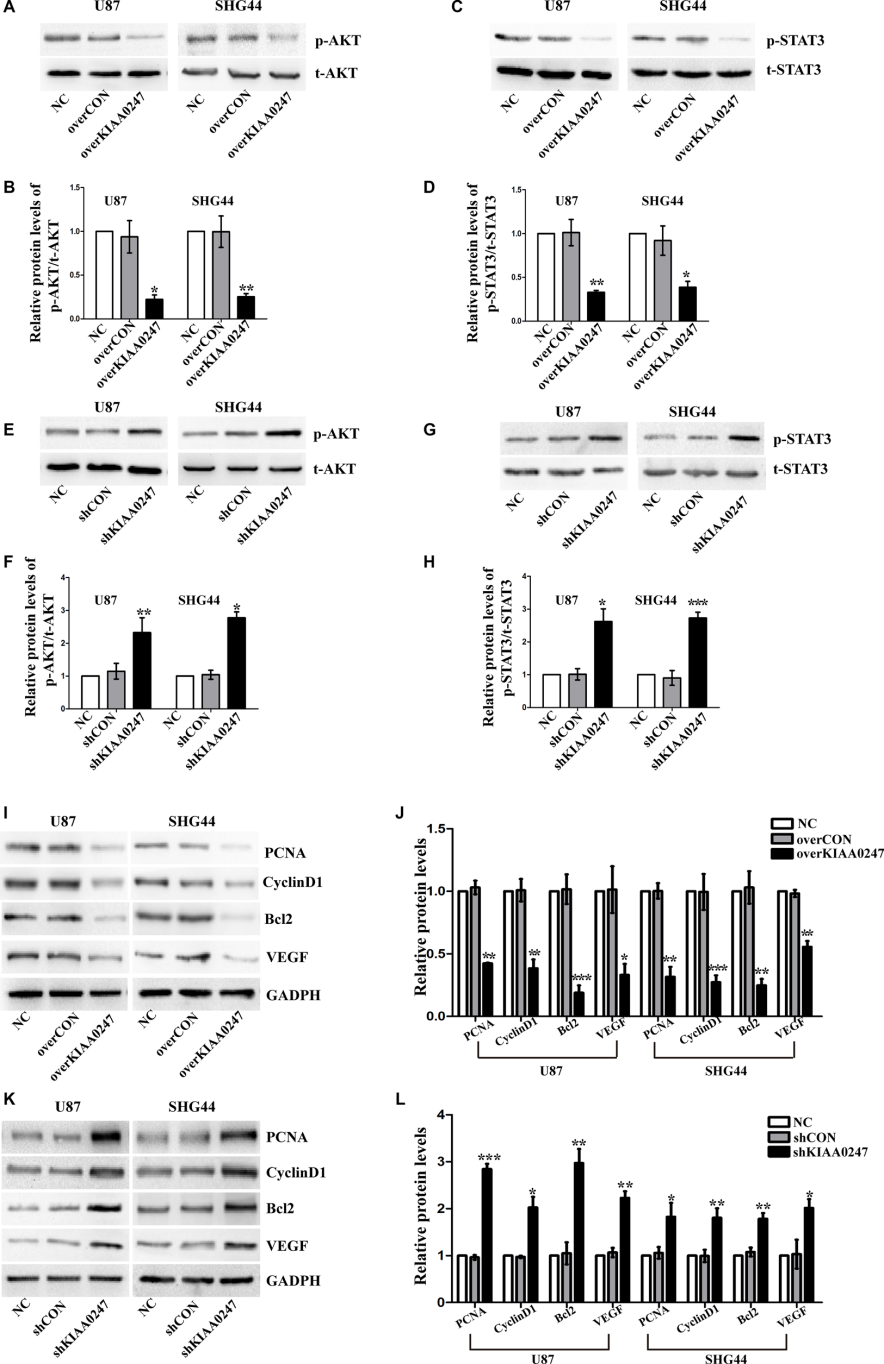
KIAA0247 suppressed AKT signaling and STAT3 signaling in glioma cells (**A**–**D**) KIAA0247 overexpression inhibits AKT and STAT3 signaling pathways. (**E**–**H**) Knockdown of KIAA0247 enhances AKT and STAT3 signaling pathways. (**I**–**L**) U87 and SHG44 cells with overexpression or knockdown of KIAA0247 were used to analyze the expression of PCNA, CyclinD1, Bcl2, VEGF. Data were based on at least 3 independent experiments, and shown as the mean ± SD. (*P < 0.05, **P < 0.01, ***P < 0.001, compared with NC).

Previous studies have revealed that AKT pathway and STAT3 pathway played a crucial role in various cell functions, such as proliferation, angiogenesis, apoptosis and survival in glioma [19–24]. To further explore the molecular mechanism of KIAA0247 inhibits growth, apoptosis and angiogenesis of glioma cells. We next explored whether these two signaling pathways were affected by KIAA0247 in glioma cells, and their downstream factors related to cell growth, apoptosis and angiogenesis were detected by Western blotting. We found that the phosphorylation of AKT and STAT3 was robustly inhibited by KIAA0247 overexpression in glioma cells (Figure 6A–6D). In contrast, knockdown of KIAA0247 could enhance phosphorylation of AKT and STAT3 (Figure 6E–6H). We also found overexpression of KIAA0247 suppressed expression of PCNA, CyclinD1, Bcl2, VEGF (Figure 6I and 6J). Consistently, knockdown of KIAA0247 promote the expression of PCNA, CyclinD1, Bcl2, VEGF (Figure 6K and 6L). These data, therefore, indicated that KIAA0247 is able to inhibit AKT and STAT3 signaling cascades, likely explaining its negative effects on cell proliferation and angiogenesis and positive effects on apoptosis.

**Figure 7:**
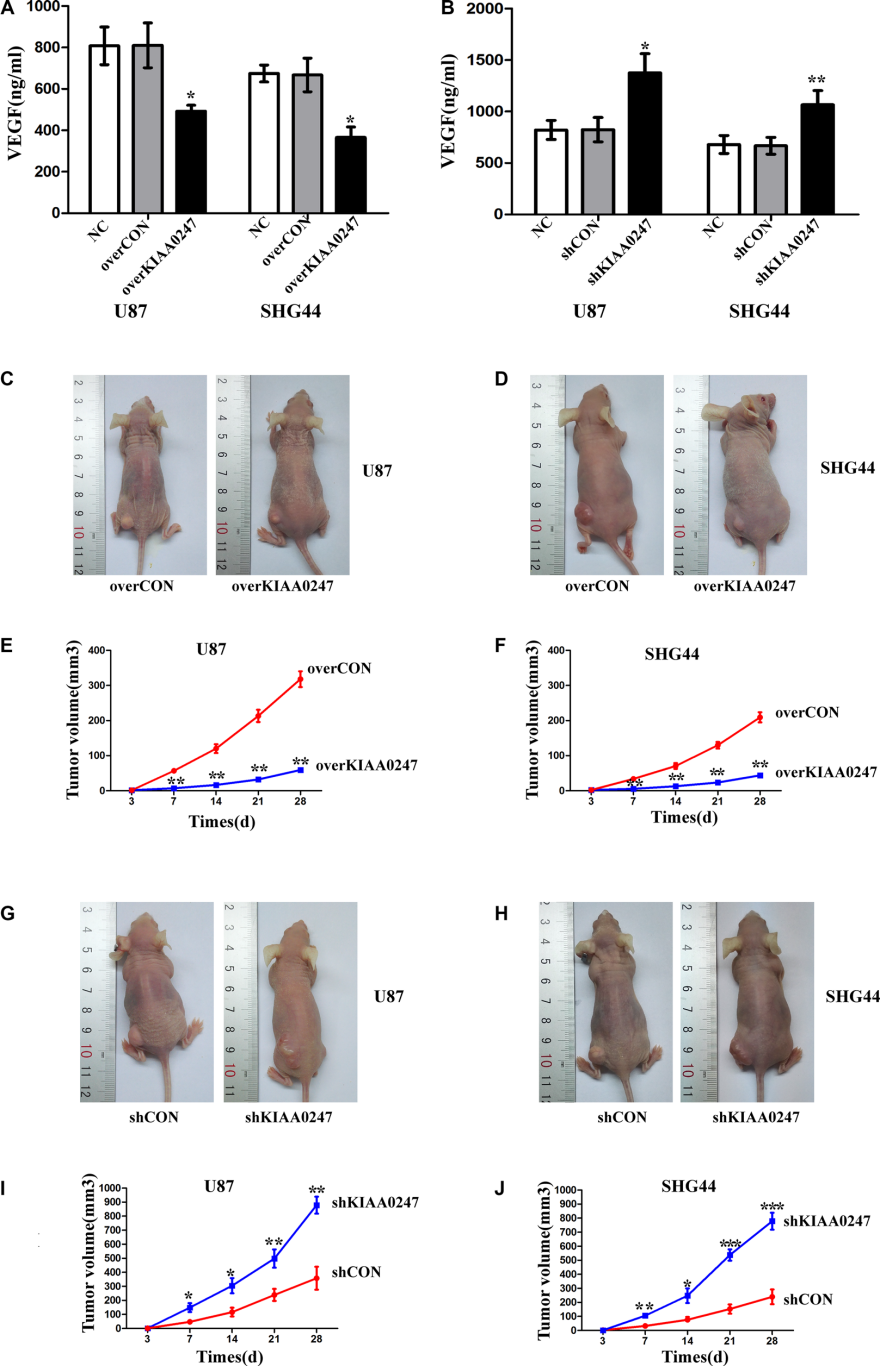
KIAA0247 overexpression reduces the growth of glioma cell xenografts in nude mice (**A**, **B**) ELISA assay shows the level of secreted VEGF in U87 and SHG44. (**C**, **D**) Representative images of subcutaneous tumor xenografts in nude mice inoculated with U87 and SHG44 cells expressing empty vectors or KIAA0247. (**E**, **F**) Time course analysis of tumor growth after injection. (**G**, **H**) Representative images of subcutaneous tumor xenografts in nude mice inoculated with U87 and SHG44 cells expressing control shRNA or KIAA0247-specific shRNA. (**I**, **J**) Time course analysis of tumor growth after injection. Data were based on at least 3 independent experiments, and shown as the mean ± SD. (*P < 0.05, **P < 0.01, ***P < 0.001 compared with NC).

VEGF expression in culture supernatants of tumor cell lines was detected by ELISA assay for the three groups. Our results demonstrated that VEGF expression was significantly lower in overKIAA0247 cells group compared with the controls (Figure 7A). In contrast, VEGF expression in shKIAA0247 cells group was higher than that in the controls (Figure 7B).

### KIAA0247 suppresses the tumor formation in xenograft model

To further investigate the effects of KIAA0247 in glioma cell growth *in vivo*, we next investigated the effects of KIAA0247 on tumor growth using a xenograft model. Glioma cells with stable expression of either control vector or KIAA0247 overexpression were implanted into the nude mice. The mice were sacrificed in 28 days when the tumor formation became obvious. The growth of the glioma cells in the mice as measured by tumor volume was significantly reduced by KIAA0247 overexpression (Figure 7C–7F). We also investigated the effect of KIAA0247 knockdown on tumor growth *in vivo*. The tumor volume was all increased by KIAA0247 knockdown in the mice (Figure 7G–7J). These observations, therefore, clearly demonstrated that KIAA0247 has a powerful activity to suppress tumorigenicity of glioma *in vivo*.

## DISCUSSION

By sequencing human cDNA clones, KIAA0201 to KIAA0280 that have critical roles in maintenance of apoptosis, cell development, and cell-to-cell interaction was determined and named [29]. Similar to the Bcl2 gene family, KIAA0271 was considered to be important in cell apoptosis [30]. Recent studies showed that KIAA0208 and KIAA0246 are involved in cell development by mediating cell fate decisions [31, 32]. KIAA0247 could increase population of the G2/M cell cycle in colorectal cancer cells and low KIAA0247 RNA level in feces was strongly correlated with colorectal tumor size and a poor survival time [9]. However, the function of KIAA0247 in glioma and its clinical significance have not yet been elucidated.

To search the possible biological functions of KIAA0247 in glioma, we tested the expression of KIAA0247 in glioma tissues at the protein and mRNA level, indicating that KIAA0247 was markedly downregulated in glioma tissues. Furthermore, KIAA0247 expression in high-grade glioma was significantly lower than in low-grade glioma. Previous study showed that, as a tumor suppressor, KIAA0247 RNA levels was deregulated in feces and correlated with a poor overall survival time in colorectal cancer patients [9]. To further elucidate the potential biological roles of KIAA0247 in glioma, we evaluated the effect of KIAA0247 overexpression on glioma cell proliferation potential by CCK8. As a result, the overexpression of KIAA0247 suppressed cell proliferation in U87 and SHG44 cell lines, whereas KIAA0247 silencing promoted cell proliferation in U87 and SHG44 cell lines. Flow cytometry indicated that induction of apoptosis and cell cycle arrest by KIAA0247 may contribute to the tumor suppressive function. Our research found KIAA0247 overexpression blocked glioma cell cycle in G1 phase, as a result of which, the glioma proliferation were inhibited by KIAA0247. Tube-formation assays indicated KIAA0247 overexpression reduced angiogenesis in tumors. In addition, a nude mice model *in vivo* revealed that the overexpression of KIAA0247 significantly reduced the capability of glioma cells to induce tumorigenesis.

Earlier studies have demonstrated that STAT3 has play an important role in regulating cell proliferation, angiogenesis, apoptosis, inflammation, oncogenesis and differentiation in tumors [19–24]. Moreover, AKT pathway has also been reported to have an important role in various cell functions in glioma [25]. Therefore, we examined whether KIAA0247 can regulate p-STAT3 and p-AKT expression. We found that the phosphorylation of AKT and STAT3 was robustly inhibited by KIAA0247 overexpression in glioma cells, in contrast, knockdown of KIAA0247 could enhance phosphorylation of AKT and STAT3, indicating that KIAA0247 can suppress cell proliferation, angiogenesis and promote apoptosis in part through AKT and STAT3 signaling pathways. In addition, AKT and STAT3 has been confirmed to be involved in the regulation of downstream factors including PCNA [33], Bcl2 [34, 35], CyclinD1 [33, 35] and VEGF [36, 37], which are related to tumor proliferation, apoptosis and angiogenesis in glioma cells. Furthermore, we found that KIAA0247 overexpression suppressed cell growth and angiogenesis and promote apoptosis through downregulation of PNCA, CyclinD1, Bcl-2 and VEGF expression and upregulation of p-AKT and p-STAT3. These findings suggested that KIAA0247 might function as a tumor suppressor in glioma cells via inhibition of the AKT and STAT3 signaling. Schwanzer-Pfeiffer D et al. reported that the low KIAA0247 level induced a downexpression of CCL2 in response to lipopolysaccharides treatment *in vitro* [38] and upregulation of CCL2 promotes glioma growth [39]. Furthermore, CCL2 increases Akt and Stat3 phosphorylation [40–43]. Therefore, we speculate that CCL2 may participate in the process of KIAA0247-induced inactivation of AKT and Stat3. It remains unclear whether there is cross-talk between the AKT and STAT3 signaling pathways or they work independently to promote progression of glioma. In the further studies, we need to explore the association between these two signaling pathways in the context of KIAA0247-inhibited glioma progression.

Our present findings confirm that KIAA0247 expression is frequently decreased in glioma tissue, KIAA0247 overexpression inhibits cell growth and angiogenesis and promotes apoptosis *in vitro* and *in vivo* via inhibition of AKT and STAT3 signaling. These results demonstrate that KIAA0247 plays a crucial role in glioma and finding out the underlying mechanism could be a promising strategy for the treatment of glioma.

## MATERIALS AND METHODS

### Cell culture and reagents

SHG44, U87, U251, and A172 glioma cell lines were from Shanghai Life Academy of Sciences Cell Library. The four glioma cell lines were maintained in a 5% CO2 atmosphere at 37°C in DMEM supplemented with 100 U/ml penicillin, 100 mg/ml streptomycin (Hyclone) and 10% FBS. Antibodies against KIAA0247 were obtained from Sigma Aldrich, AKT, phospho-AKT(Thr308), phospho-STAT3 (Tyr705), STAT3, PCNA, Bcl2, CyclinD1, VEGF were obtained from Cell Signaling Techology. GAPDH was purchased from KangCheng Biotech.

### Patients and tissue preparation

A total of 112 glioma samples and 11 non-tumor brain samples were obtained from between 2006 and 2013 in the First and Second Affiliated Hospitals of Chongqing Medical University. Additionally, eight astrocytomas and the corresponding adjacent non-cancerous brain tissues were collected in 2015. None of the patients had received prior chemotherapy or radiotherapy. The patients' clinical characteristics such as age, gender, and WHO grade, were collected for statistical analysis. The patients' prognoses were obtained from clinical services. Patients' consent and approval from the Institutional Research Ethics Committee of Chongqing Medical University were obtained for research purposes. For histological analysis, resected glioma and non-neoplastic brain tissues were fixed in formalin, embedded in paraffin and cut into 5-μm thick sections. For qRT-PCR and western blot analysis, tissues were immediately frozen in liquid nitrogen and kept at –80°C until analysis.

### Immunohistochemistry

Tissue sections were cut and mounted on slides. After de-waxing and rehydration, the sections were antigen-retrieved in 10 mm citrate buffer for 5 min at 100°C. Endogenous peroxidase activity and non-specific antigens were blocked with 3% hydrogen peroxide and serum, followed by incubation with KIAA0247 antibody overnight at 4°C. Slides were then incubated with goat anti-rabbit secondary antibody, developed using 3,3-diaminobenzidine (DAB) solution and counterstained with hematoxylin. PBS was used in place of the primary antibodies for the negative controls which were processed along with the samples. No apparent immunoreactivity was detected in negative controls. After staining, the slides were reviewed by two independent pathologists using a microscope (DM6000 B; Leica, Wetzlar, Germany). Immunohistochemical staining of KIAA0247 was calculated as both percentage of positive cells and color intensity. The percentage of the positivity was classified as “0” (negative), “1” (< 10%), “2” (10–50%), and “3” (> 50%). The intensity was graded as “0” (absent), “1” (light yellow), “2” (yellowish brown), and “3” (brown). The expression of KIAA0247 was evaluated by staining index (SI), which was calculated using the following formula: SI = proportion score×intensity score. SI of 0 was categorized as negative (–), 1–2 as low expression (1+), 3–4 as moderate expression (2+), 6 or 9 as high expression (3+).

### KIAA0247 knockdown and overexpression in glioma cells by lentivirus particles

KIAA0247 lentivirus (overKIAA0247) used for KIAA0247 overexpression and corresponding negative control lentivirus (overCON), as well as lentiviral constructs expressing KIAA0247 shRNA (shKIAA0247) and matched empty lentivirus (shCON) were purchased from Genechem Co., Ltd. Glioma cells stably expressing the KIAA0247 shRNA targeting the sequence CATACGAGGAGGCTGTATATG. One day before transfection, 5 × 10^4^cells per well (reaching about 30% confluency at the time of transfection) were cultured in six-well plates. These lentivirus were introduced into glioma cells treated with 8 ug/ml polybrene (Genechem) and complete medium. Transfection effects were observed by a fluorescence microscope after 48 h. Puromycin was used to purify these infected cells. Effective knockdown or forced expression of KIAA0247 was monitored by real time polymerase chain reaction (PCR) and western blot analysis after 72 h.

### Real-time PCR

Total RNA in cells and tissues were extracted using RNAiso Plus (TaKaRa). The concentrations of these RNA samples were then measured using a spectrophotometer and the RNA specimens were reverse-transcribed into cDNA using the Primescript RT reagent Kit (TaKaRa). The primer sequence and product size for KIAA0247 was: forward 5′-CTGCAGATTCAGAGAACAGTGAC-3′ and reverse 5′-CTCATGCTTCTTTCAACAGTGG-3′, 93 bp, and for GAPDH, which was used as a standard, was: forward 5′-CTTTGGTATCGTGGAAGGACTC-3′ and reverse 5′-GTAGAGGCAGGGATGATGTTCT-3′, 132 bp. Amplification conditions were as follows: 95°C for 30 s, followed by 40 cycles at 95°C for 15s, and 60°C for 45s. The relative fold-changes in mRNA levels were calculated according to the 2^−ΔΔCT^ method [44].

### Western blot

The tissues and cells were lysed by RIPA Lysis Buffer (Beyotime Institute of Biotechnology, Beijing, China) containing PMSF and phosphatase inhibitor. An equal amount of each protein sample was separated by 8–12% SDS–PAGE and transferred onto PVDF membranes. After incubation with primary antibodies overnight at 4°C including KIAA0247 (1:1000), total AKT1 (1:1000), phospho-AKT (1:250), total STAT3 (1:500), phospho-STAT3 (1:500), GAPDH (1:800), PCNA (1:500) and CyclinD1 (1:500), VEGF (1:500), Bcl2 (1:500), the PVDF membranes were washed 3 times with TBST buffer, and incubated with secondary antibody (1:5000) for 1h at 37°C. Then the membranes were washed 3 times in TBST buffer and the amount of protein in each band was quantified using the Quantity One 4.6 computer software (Bio-Rad, Hercules, California).

### Cell proliferation assay

Cells were seeded in 96-well plates at a density of 2000 cells/well. For 24, 48, 72, and 96 h, 10 ul of Cell Counting Kit-8 (Beyotime Institute of Biotechnology) was added to each well and the cells were incubated for another 1 h. The absorbance values were read at 450 nm using an enzyme-labeled instrument.

### Flow cytometric assay to detect cell cycle and apoptosis

5 × 10^5^ cells were harvested and fixed in 70% icecold ethanol overnight. Then cells were incubated with 10 mg/ml RNase (Sigma and 50 mg/ml propidium iodide (Sigma) at 37°C for 30 min in the dark. The cell cycle was analyzed by flow cytometry (BD Bioscience).

To determine cell apoptosis, the same-treated cells were harvested and incubated with reagents from the Annexin V-FITC apoptosis kit (BioVision) according to the manufacturer's protocol.

### ELISA for VEGF in cell culture supernatants

VEGF concentration in supernatants of the cultured glioma cells was determined using commercial human VEGF ELISA kit according to the manufacturer's instructions (R&D System).

### Tubule formation assay

HUVECs were plated in Matrigel (BD Science) precoated 48-well plate at 2 × 10^3^ cells/well with conditioned medium from NC, overCON, overKIAA0247, shCON or shKIAA0247 glioma cells. Images were captured under bright-fields using a microscope (DM6000 B; Leica, Wetzlar, Germany). The tubule crosses were quantified by Image J software at three random 10× fields to get the sum. The experiment was repeated three times.

### Xenograft tumor model

The male nude mice (4 wk old) used in this study were provided by experimental animal center of Chongqing Medical University. All animal studies were approved by the Ethics Committee of Chongqing Medical University. The glioma cells were resuspended in DMEM at a density of 2 × 10^6^ cells per 50 ul and were injected subcutaneously into the nude mice. Tumor volumes were recorded at 7, 14, 21, and 28 d after inoculation according to the formula described previously [45].

### Statistical analysis

Statistical analyses were performed using SPSS 17.0. Statistical differences among groups were analyzed by *t-test* or χ^2^-square test. The prognostic significance analysis was performed using Kaplan-Meier method and log-rank tests. *P* < 0.05 was considered to be statistically significant. All data are presented as mean ± standard deviation (SD).

## SUPPLEMENTARY MATERIALS TABLES


