# Supplementary material for: KIAA0247 suppresses the proliferation, angiogenesis and promote apoptosis of human glioma through inactivation of the AKT and Stat3 signaling pathway
Source: Oncotarget. 2016 Nov 23;7(52):87100–13. doi: 10.18632/oncotarget.13527 (PMC5349974; doi:10.18632/oncotarget.13527)
Supplement: Supplementary file 1 [file oncotarget-07-87100-s001.pdf]

KIAA0247 suppresses the proliferation, angiogenesis and promote apoptosis of human glioma through inactivation of the AKT and Stat3 signaling pathway

Supplementary Materials

Supplementary Table S1: Clinical Characteristics of 112 Patients and 11 Non-Tumor Specimens

	n	%
Gender		
Male	59	47.97
Female	64	52.03
Age(years)		
> 45	63	51.22
≤ 45	60	48.78
Glioma histology		
WHO grade		
Non-tumor	11	8.94
I	16	13.01
II	38	30.89
III	32	26.02
IV	26	21.14

Supplementary Table S2: Correlation expression of KIAA0247 in glioma tissues and normal human brain tissues

Groups	Number	KIAA0247 expression				*P*
		–	1+	2+	3+	
Tissue						
Normal tissue	11	0	0	2	9	0.000
Glioma tissue	112	29	36	40	7	
Gender						
Male	58	14	20	22	2	0.567
Female	54	15	16	18	5	
Age (yeas)						
> 45	57	14	19	19	5	0.653
≤ 45	55	15	17	21	2	
WHO grade						
I–II	54	3	17	27	7	0.000
III–IV	58	26	19	13	0	

Supplementary Table S3: Clinical Characteristics of Eight Glioma Patients and KIAA0247 Protein Expression in Tumor Tissues Compared With Their Matched Adjacent Non-Tumorous Brain Tissues

				KIAA0247/GADPH (protein expression)	
				N	**T**
Number	Age (years)	Gender	Grade		
1	55	Female	I	1.24 ± 0.04	0.66 ± 0.11**
2	42	Male	II	1.45 ± 0.11	0.56 ± 0.08**
3	36	Female	II	0.66 ± 0.01	0.55 ± 0.07
4	46	Female	III	1.30 ± 0.14	0.50 ± 0.13**
5	38	Male	III	1.73 ± 0.14	0.63 ± 0.11**
6	29	Female	III	2.20 ± 0.30	0.54 ± 0.04*
7	44	Male	IV	2.04 ± 0.18	0.71 ± 0.05**
8	61	Female	IV	1.46 ± 0.05	0.65 ± 0.06***
